# Revisiting the relationship between the big five personality traits and flow using meta-analytic structural equation modeling: a methodological perspective on Buseyne et al. (2025)

**DOI:** 10.3389/fpsyg.2026.1759068

**Published:** 2026-02-25

**Authors:** Jie Liu, Junhua Dang, Xiaoyu Liu, Huiqin Yang

**Affiliations:** 1 School of Humanities and Social Sciences, Xi'an Jiaotong University, Xi'an, China; 2 Department of Surgical Sciences, Uppsala University, Uppsala, Sweden

**Keywords:** big five personality traits, flow, inter-trait correlations, meta-analytic structural equation modeling (MASEM), personality-flow associations

## 1 Introduction

Flow is a state of optimal experience characterized by deep concentration, immersion, and intrinsic enjoyment, and it is widely regarded as a key psychological mechanism that promotes learning efficiency, creativity, performance, and well-being (Csikszentmihalyi, 1990). In recent years, increasing attention has been given to individual differences such as “flow proneness” and the “autotelic personality”. Empirical studies have consistently shown that individuals differ in their likelihood of experiencing flow in everyday contexts, and these differences are closely linked to the Big Five personality traits (Baumann, 2021; Tse et al., 2021; Ullén et al., 2016).

Against this backdrop, Buseyne et al. (2025) published the most comprehensive three-level meta-analysis to date in the Journal of Personality, synthesizing 352 effect sizes from 24 studies to examine the associations between the Big Five traits and flow, and to evaluate potential moderators including culture, context, flow dimensions, age, gender, and measurement tools. Their findings indicated that conscientiousness showed the strongest positive relation with flow, followed by extraversion, openness, and agreeableness, whereas neuroticism showed a negative relation. They also reported that the effects of extraversion, openness, and agreeableness were stronger in Eastern cultures, and that the magnitude of effects varied across flow measures.

Although these findings provide important evidence concerning personality-flow relations, traditional meta-analytic approaches based on zero-order correlations carry methodological limitations. In practice, these approaches implicitly assume that each personality trait operates independently, treating each “trait–flow” correlation as a statistically independent unit. However, decades of personality research demonstrate that the Big Five traits exhibit systematic and nontrivial intercorrelations in empirical data. Despite being orthogonal by design at the theoretical level, they frequently show stable internal correlations across self-report measures, interview data, and cross-cultural samples–correlations that may arise from common method variance, higher-order factors such as the Alpha/Beta factors, or even a general personality factor (GFP; Digman, 1997; Musek, 2007; van der Linden et al., 2010).

Because of this inherent structural covariance, zero-order correlations inevitably conflate unique trait effects with shared variance, potentially inflating the apparent direct contribution of certain traits to flow and reducing the precision of theoretical interpretations. This raises a core question: To what extent do the personality-flow correlations observed in traditional meta-analyses reflect the unique predictive power of each trait, rather than artifacts of inter-trait collinearity? In other words, can zero-order correlations meaningfully support interpretations of “independent” trait effects when the traits themselves are highly correlated?

With the advancement of Meta-Analytic Structural Equation Modeling (MASEM), scholars have increasingly emphasized the importance of explicitly modeling effect-size dependency and intercorrelations in meta-analysis (Busseri and Erb, 2024; Jak and Cheung, 2020; Steinmetz and Block, 2022). In this context, we argue that research on personality-flow relations should adopt analytic frameworks capable of modeling multiple predictors simultaneously while incorporating their internal correlation structure. MASEM provides a powerful approach for estimating the unique contributions of the Big Five traits with greater accuracy and theoretical clarity.

## 2 Reanalysis

A key strength of MASEM is its ability to incorporate the correlational structure among variables into effect-size synthesis, thereby enabling simultaneous estimation of multivariate relations (Jak and Cheung, 2018; Wilson et al., 2016). In its standard two-stage procedure, Stage 1 synthesizes the correlation matrices across studies to obtain a weighted pooled correlation matrix, and Stage 2 fits a structural equation model to this matrix to estimate the “unique effects” of multiple predictors on an outcome. Importantly, the Stage-1 estimation is conducted using a three-level multilevel model, which accounts for statistical dependencies among multiple effect sizes extracted from the same study and ensures more robust estimation of the pooled correlation matrix (Wilson et al., 2016). For the Big Five–flow relationship, this approach allows all five “trait → flow” paths to be estimated within a single model, while explicitly incorporating intercorrelations among the traits. As a result, MASEM effectively addresses inter-trait collinearity and avoids interpretational pitfalls associated with zero-order correlations. Because the Stage-2 MASEM model is just-identified (*df* = 0) and reproduces the pooled Stage-1 correlation matrix by construction, global fit indices are reported for completeness but are not informative for assessing overall model fit (χ^2^ = 0, RMSEA = 0, SRMR = 0, CFI = 1; TLI is undefined under *df* = 0). Accordingly, inference focuses on the estimated path coefficients and their confidence intervals.

In the present study, we used the database made publicly available by Buseyne et al. (2025) and supplemented missing trait intercorrelations by retrieving original articles and contacting study authors. All 24 included studies provided zero-order correlations between the Big Five traits and flow and were therefore included in the Stage-1 estimation of the trait–flow associations. Although only 13 of the 24 studies reported complete intercorrelations among all five personality traits, these data were used to estimate the pooled inter-trait correlation structure required for the multivariate model. We conducted a two-stage MASEM in which flow served as the outcome and the Big Five as simultaneous predictors, estimating all five regression paths along with their variance–covariance matrix using maximum likelihood estimation. All analyses were implemented using R and the metaSEM and OpenMx packages. The data and code supporting our findings are made available in a repository (https://osf.io/z4anb/files).

### 2.1 Re-examining personality-flow relations

**Figure 1:**
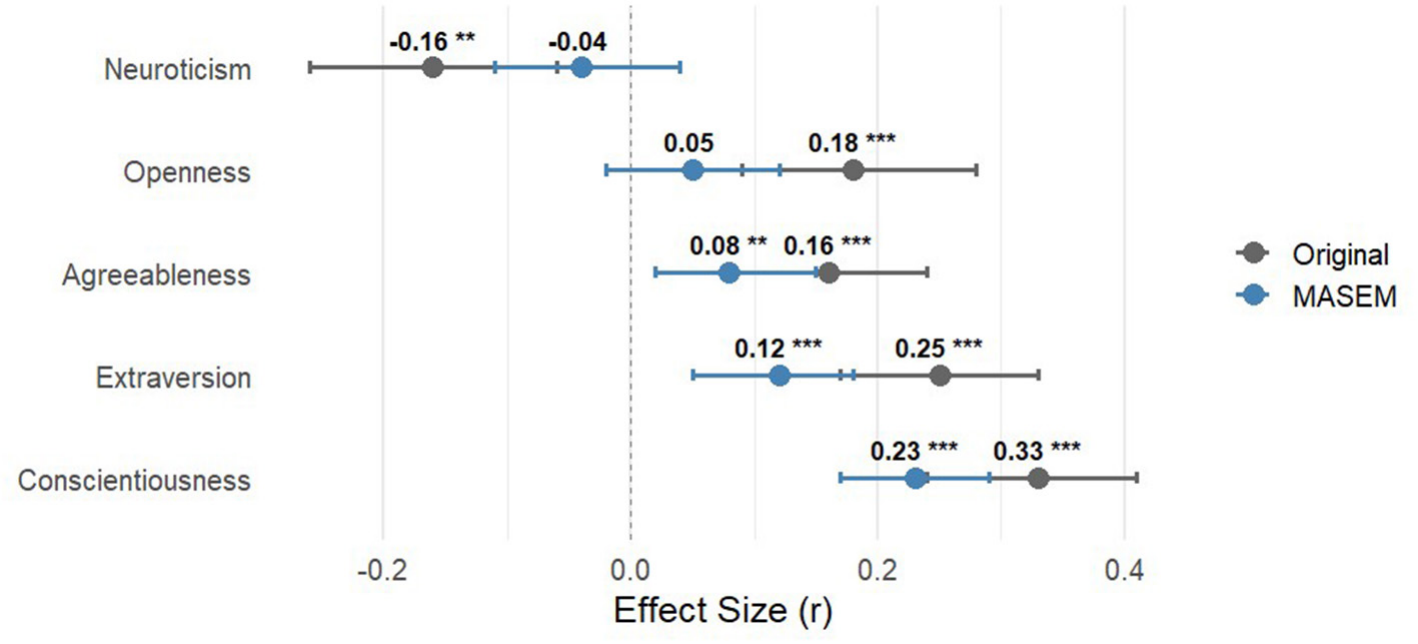
Summary of Buseyne et al. (2025) results and the current MASEM estimates. Error bars represent 95% confidence intervals. Asterisks indicate statistical significance for both Original and MASEM estimates (**p < 0.01; ***p < 0.001). No asterisk indicates *p* ≥ 0.05.

As shown in the pooled correlation matrix (Supplementary Table S1), the Big Five traits exhibit moderate intercorrelations, indicating substantial shared variance that is not accounted for in zero-order associations. The reanalysis revealed a systematic shift in the personality-flow association patterns once inter-trait collinearity was controlled. All traits showed attenuated effects compared to zero-order meta-analytic results (Figure 1). Conscientiousness decreased from *r* = 0.33 to *r* = 0.23, remaining significant and robust, and emerged as the only trait with a clear predictive effect under the multivariate framework. Extraversion (0.25 → 0.12) and agreeableness (0.16 → 0.08) showed substantially reduced predictive effects, while openness (0.18 → 0.05) and neuroticism (−0.16 → −0.04) became non-significant after adjusting for other traits.

This consistent attenuation suggests that traditional zero-order correlations may systematically overestimate the direct contribution of openness, extraversion, agreeableness, and neuroticism to flow. Much of their apparent effect likely reflects shared variance with other traits—particularly conscientiousness (Buecker et al., 2020; Van Der Linden et al., 2010). In contrast, the central role of conscientiousness became more pronounced in the multivariate model, positioning it as the primary personality basis for individual differences in flow.

### 2.2 Reassessing moderating effects

To evaluate whether personality-flow relations vary across study characteristics, we examined all study-level moderators included in Buseyne et al. (2025): gender, age, culture, research context (education vs. work vs. sports vs. music vs. other), flow measurement instruments, and personality measurement instruments. Because MASEM can only incorporate study-level moderators, within-study flow dimensions were excluded. Gender was recoded into three groups based on the percentage of female participants (< 40%, 40–60%, >60%), and age was divided into four equal intervals based on sample mean age (10–17, 18–25, 26–33, 34–41). Because the youngest age group lacked complete Big Five intercorrelations, it was excluded. Similarly, IPIP-based measures were not included in the personality-instrument moderator analysis due to missing inter-trait correlations.

All moderator variables were examined using the same multigroup MASEM procedure. First, we conducted Stage-1 meta-analyses separately within each subgroup, using a three-level random-effects model to synthesize all correlation coefficients. The resulting vectors of pooled correlations and their asymptotic covariance matrices were then reassembled into 6 × 6 correlation matrices, which served as input for Stage 2. Next, we fitted the same structural equation model within each subgroup, allowing the five personality traits to intercorrelate freely while jointly predicting flow. Finally, moderation was tested through multigroup model comparison: the unconstrained model freely estimated all trait → flow paths across groups, whereas the constrained model imposed equality constraints on these five paths. A significant deterioration in model fit under the constraints was taken as evidence of moderation.

Based on the unified multigroup MASEM procedure described above, the moderation analyses largely replicated the conclusions of the original study. For gender [Δχ^2^(10) = 8.323, *p* = 0.597], age [Δχ^2^(10) = 5.365, *p* = 0.865], research context [Δχ^2^(20) = 31.322, *p* = 0.051], and personality measurement instruments [Δχ^2^(10) = 14.186, *p* = 0.165], the model comparisons revealed no significant differences, indicating that the structural paths from the Big Five traits to flow were highly consistent across subgroups and were not influenced by these study characteristics. However, two moderators previously identified as significant in Buseyne et al. (2025)—culture and flow measurement instruments—displayed different patterns in our reanalysis.

#### 2.2.1 Culture

Although Buseyne et al. reported stronger effects of extraversion, openness, and agreeableness in Eastern samples, these cultural differences vanished entirely in MASEM after accounting for the internal correlation structure of the Big Five [Δχ^2^ (5) = 4.206, *p* = 0.520]. This suggests that the cultural differences observed at the zero-order level likely stem from culturally patterned inter-trait covariance rather than from genuine variation in the unique effects of specific traits. For example, cross-cultural research indicates that agreeableness and conscientiousness are more strongly correlated in collectivistic cultures due to the emphasis on interpersonal harmony and role obligations (Church et al., 2011; Yoon et al., 2002). Such heightened covariation can inflate zero-order personality-flow associations, and once shared variance is partitioned out, cultural differences disappear.

#### 2.2.2 Flow measurement instruments

Flow measurement instruments demonstrated some degree of moderation, yet the effects were limited to fewer paths and were noticeably attenuated. The omnibus test for the Dispositional Flow Scale (DFS), the Flow State Scale (FSS), the Swedish Flow Proneness Questionnaire (SFPQ), and other scales was significant [Δχ^2^(15) = 71.675, *p* < 0.001], indicating at least one trait → flow path differed across instruments. Follow-up path-specific tests revealed that only the conscientiousness → flow path remained significant after Holm correction (*p*_holm = 0.040). Pairwise comparisons identified a significant difference between FSS and SFPQ (*p*_holm = 0.031). Conceptually, the FSS—designed to assess state flow—emphasizes “clear goals” and “sense of control” (Jackson and Eklund, 2002), components closely tied to the goal-directed and self-regulatory characteristics of conscientiousness. In contrast, the SFPQ, a brief 10-item trait-like flow proneness scale, focuses on core experiential aspects of flow (e.g., automaticity, enjoyment; Ullén et al., 2012), which are less aligned with conscientiousness-specific processes. Measurement research further suggests that shorter scales with narrower construct coverage may have reduced sensitivity to broad personality dimensions (Credé et al., 2012), providing additional support for this pattern.

Overall, these findings highlight that moderator effects attributed to “cultural context” or “flow measurement instruments differences” in traditional meta-analyses may largely reflect variation in the internal correlational structure of personality traits, rather than meaningful changes in the substantive relations between personality and flow. This underscores the importance of controlling inter-trait correlations when evaluating the robustness of trait-flow associations and demonstrates the theoretical and methodological advantages of MASEM over traditional element wise meta-analytic approaches.

## 3 Discussion

Introducing MASEM into the study of personality and flow provides a more rigorous and theoretically consistent lens to conceptualize their relationship. Unlike traditional meta-analytic approaches, which independently examine each zero-order correlation, MASEM reveals a more coherent structural pattern: conscientiousness stands out as the primary and robust predictor of flow, whereas the apparent effects of other traits largely dissipate once shared variance is controlled. This refined understanding clarifies that flow experiences are not simply driven by high extraversion, low neuroticism, or high openness *per se*, but may stem from a deeper underlying capacity for sustained attention, goal regulation, and task engagement characteristic of conscientious individuals.

The present findings also suggest that previously reported cultural moderation effects may reflect differences in overall personality structure rather than genuine cross-cultural variation in specific trait effects. Nevertheless, the limited availability of full Big Five correlation matrices across studies warrants caution, and future work should broaden data coverage to strengthen the robustness of these conclusions.

At the theoretical level, this methodological shift prompts a reconsideration of long-standing assumptions about the role of personality in flow. Traits such as openness and extraversion may operate more as “background traits” that create a conducive experiential environment rather than as direct causal antecedents of flow. From a methodological standpoint, the use of MASEM highlights the need for greater transparency and data sharing, particularly regarding the reporting of full covariance or correlation matrices. As more studies adopt comprehensive reporting practices, the application of MASEM and related multivariate meta-analytic techniques will become increasingly feasible, facilitating more precise theoretical development in complex psychological domains.

In conclusion, this study demonstrates that a multivariate analytic framework yields more accurate and theoretically coherent insights than traditional univariate meta-analyses. Conscientiousness appears to be the key personality foundation of flow, whereas the influence of other traits becomes substantially weaker once shared variance is accounted for. These findings not only refine the conclusions of Buseyne et al. (2025) but also highlight the necessity of using MASEM in investigating multivariate psychological phenomena.

## References

[B1] Baumann N. (2021). “Autotelic personality,” in Advances in Flow Research, eds. C. Peifer, and S. Engeser (Springer International Publishing), 231–261. doi: 10.1007/978-3-030-53468-4_9

[B2] Buecker S. Maes M. Denissen J. J. A. Luhmann M. (2020). Loneliness and the big five personality traits: a meta-analysis. Eur. J. Pers. 34, 8–28. doi: 10.1002/per.2229

[B3] Buseyne S. Said-Metwaly S. Van den Noortgate W. Depaepe F. Raes A. (2025). The relationship between personality and flow: a meta-analysis. J. Pers. doi: 10.1111/jopy.70004. [Epub ahead of print]. 40579783 PMC12988346

[B4] Busseri M. A. Erb E. M. (2024). The happy personality revisited: re-examining associations between Big Five personality traits and subjective well-being using meta-analytic structural equation modeling. J. Pers. 92, 968–984. doi: 10.1111/jopy.12862 37462061

[B5] Church A. T. Alvarez J. M. Mai N. T. Q. French B. F. Katigbak M. S. Ortiz F. A. (2011). Are cross-cultural comparisons of personality profiles meaningful? Differential item and facet functioning in the revised NEO personality inventory. J. Pers. Soc. Psychol. 101, 1068–1089. doi: 10.1037/a0025290 21910552

[B6] Credé M. Harms P. Niehorster S. Gaye-Valentine A. (2012). An evaluation of the consequences of using short measures of the Big Five personality traits. J. Pers. Soc. Psychol. 102, 874–888. doi: 10.1037/a0027403 22352328

[B7] Csikszentmihalyi M. (1990). Flow: The Psychology of Optimal Experience. New York, NY: Harper and Row.

[B8] Digman J. M. (1997). Higher-order factors of the Big Five. J. Pers. Soc. Psychol. 73, 1246–1256. doi: 10.1037/0022-3514.73.6.1246 9418278

[B9] Jackson S. A. Eklund R. C. (2002). Assessing flow in physical activity: the flow state scale-2 and dispositional flow scale-2. J. Sport Exerc. Psychol. 24, 133–150. doi: 10.1123/jsep.24.2.133

[B10] Jak S. Cheung M. W.-L. (2018). Testing moderator hypotheses in meta-analytic structural equation modeling using subgroup analysis. Behav. Res. Methods 50, 1359–1373. doi: 10.3758/s13428-018-1046-3 29869223 PMC6096661

[B11] Jak S. Cheung M. W. -L. (2020). Meta-analytic structural equation modeling with moderating effects on SEM parameters. Psychol. Methods 25, 430–455. doi: 10.1037/met0000245 31670537

[B12] Musek J. (2007). A general factor of personality: evidence for the big one in the five-factor model. J. Res. Pers. 41, 1213–1233. doi: 10.1016/j.jrp.2007.02.003

[B13] Steinmetz H. Block J. (2022). Meta-analytic structural equation modeling (MASEM): new tricks of the trade. Manag. Rev. Q. 72, 605–626. doi: 10.1007/s11301-022-00293-6

[B14] Tse D. C. K. Nakamura J. Csikszentmihalyi M. (2021). Living well by “flowing' well: the indirect effect of autotelic personality on well-being through flow experience. J. Posit. Psychol. 16, 310–321. doi: 10.1080/17439760.2020.1716055

[B15] Ullén F. de Manzano Ö. Almeida R. Magnusson P. K. E. Pedersen N. L. Nakamura J. . (2012). Proneness for psychological flow in everyday life: associations with personality and intelligence. Pers. Individ. Differ. 52, 167–172. doi: 10.1016/j.paid.2011.10.003

[B16] Ullén F. Harmat L. Theorell T. Madison G. (2016). “Flow and individual differences – a phenotypic analysis of data from more than 10,000 twin individuals,” in Flow Experience: Empirical Research and Applications, eds. L. Harmat, F. Ørsted Andersen, F. Ullén, J. Wright, and G. Sadlo (Springer International Publishing), 267–288. doi: 10.1007/978-3-319-28634-1_17

[B17] Van Der Linden D. Scholte R. H. J. Cillessen A. H. N. Nijenhuis J. T. Segers E. (2010). Classroom ratings of likeability and popularity are related to the Big Five and the general factor of personality. J. Res. Pers. 44, 669–672. doi: 10.1016/j.jrp.2010.08.007

[B18] van der Linden D. te Nijenhuis J. Bakker A. B. (2010). The general factor of personality: a meta-analysis of Big Five intercorrelations and a criterion-related validity study. J. Res. Pers. 44, 315–327. doi: 10.1016/j.jrp.2010.03.003

[B19] Wilson S. J. Polanin J. R. Lipsey M. W. (2016). Fitting meta-analytic structural equation models with complex datasets. Res. Synth. Methods 7, 121–139. doi: 10.1002/jrsm.1199 27286899 PMC4905597

[B20] Yoon K. Schmidt F. Ilies R. (2002). Cross-cultural construct validity of the five-factor model of personality among Korean employees. J. Cross Cult. Psychol. 33, 217–235. doi: 10.1177/0022022102033003001

