# Supplementary material for: Revisiting the relationship between the big five personality traits and flow using meta-analytic structural equation modeling: a methodological perspective on Buseyne et al. (2025)
Source: Front Psychol. 2026 Feb 25;17:1759068. doi: 10.3389/fpsyg.2026.1759068 (PMC12975761; doi:10.3389/fpsyg.2026.1759068)
Supplement: Supplementary file 1 [file Table_1.docx]

**Revisiting the Relationship Between the Big Five Personality Traits and Flow Using Meta-Analytic Structural Equation Modeling: A Methodological Perspective on Buseyne et al. (2025)**

Supplementary Table S1. Correlations between big five personality traits and flow.

| Variables | 1 | 2 | 3 | 4 | 5 | 6 |
| --- | --- | --- | --- | --- | --- | --- |
| 1. Neuroticism | - |  |  |  |  |  |
| 2. Openness | -0.13* | - |  |  |  |  |
| 3. Agreeableness | -0.20*** | 0.20*** | - |  |  |  |
| 4. Extraversion | -0.22*** | 0.26*** | 0.21*** | - |  |  |
| 5. Conscientiousness | -0.22*** | 0.23*** | 0.28*** | 0.27*** | - |  |
| 6. Flow | -0.13*** | 0.15*** | 0.19*** | 0.22*** | 0.30*** | - |

*Note*: The pooled intercorrelations among the Big Five traits are based on 13 studies that reported complete personality correlation matrices. Trait–flow correlations were synthesized using all 24 studies.

* *p* < .05; *** *p* < .001.

Supplementary Table S2. Numerical summary of Original meta-analytic correlations and MASEM standardized path estimates corresponding to Figure 1.

|  | Original | | | |  | MASEM | | | |
| --- | --- | --- | --- | --- | --- | --- | --- | --- | --- |
| Variables | Effect size (r) | SE | *95% CI* | *p* |  | Standardized path estimate | SE | *95% CI* | *p* |
| Neuroticism | -0.16 | 0.05 | [-0.26, -0.06] | 0.002 |  | -0.04 | 0.04 | [-0.11, 0.04] | 0.334 |
| Openness | 0.18 | 0.05 | [0.09, 0.28] | < 0.001 |  | 0.05 | 0.04 | [-0.02, 0.12] | 0.182 |
| Agreeableness | 0.16 | 0.04 | [0.08, 0.24] | < 0.001 |  | 0.08 | 0.03 | [0.02, 0.15] | 0.010 |
| Extraversion | 0.25 | 0.04 | [0.17, 0.33] | < 0.001 |  | 0.12 | 0.03 | [0.05, 0.18] | < 0.001 |
| Conscientiousness | 0.33 | 0.04 | [0.24, 0.41] | < 0.001 |  | 0.23 | 0.03 | [0.17, 0.29] | < 0.001 |
